# Analysis and Reduction of Nonlinear Distortion in AC-Coupled CMOS Neural Amplifiers with Tunable Cutoff Frequencies

**DOI:** 10.3390/s21093116

**Published:** 2021-04-30

**Authors:** Beata Trzpil-Jurgielewicz, Władysław Dąbrowski, Paweł Hottowy

**Affiliations:** Faculty of Physics and Applied Computer Science, AGH University of Science and Technology, 30-059 Kraków, Poland; btrzpil@agh.edu.pl (B.T.-J.); W.Dabrowski@ftj.agh.edu.pl (W.D.)

**Keywords:** CMOS neural amplifier, AC coupling, pseudoresistor, nonlinear distortion, area-efficient design

## Abstract

Integrated CMOS neural amplifiers are key elements of modern large-scale neuroelectronic interfaces. The neural amplifiers are routinely AC-coupled to electrodes to remove the DC voltage. The large resistances required for the AC coupling circuit are usually realized using MOSFETs that are nonlinear. Specifically, designs with tunable cutoff frequency of the input high‑pass filter may suffer from excessive nonlinearity, since the gate-source voltages of the transistors forming the pseudoresistors vary following the signal being amplified. Consequently, the nonlinear distortion in such circuits may be high for signal frequencies close to the cutoff frequency of the input filter. Here we propose a simple modification of the architecture of a tunable AC-coupled amplifier, in which the bias voltages *V_gs_* of the transistors forming the pseudoresistor are kept constant independently of the signal levels, what results in significantly improved linearity. Based on numerical simulations of the proposed circuit designed in 180 nm technology we analyze the Total Harmonic Distortion levels as a function of signal frequency and amplitude. We also investigate the impact of basic amplifier parameters—gain, cutoff frequency of the AC coupling circuit, and silicon area—on the distortion and noise performance. The post-layout simulations of the complete test ASIC show that the distortion is very significantly reduced at frequencies near the cutoff frequency, when compared to the commonly used circuits. The THD values are below 1.17% for signal frequencies 1 Hz–10 kHz and signal amplitudes up to 10 mV peak-to-peak. The preamplifier area is only 0.0046 mm^2^ and the noise is 8.3 µV_rms_ in the 1 Hz–10 kHz range. To our knowledge this is the first report on a CMOS neural amplifier with systematic characterization of THD across complete range of frequencies and amplitudes of neuronal signals recorded by extracellular electrodes.

## 1. Introduction

Multielectrode neural interfaces are widely used in basic neuroscience research [1,2,3,4] and for development of advanced brain-computer interfaces and neuroelectronic prostheses [5,6,7]. Taking advantage of large numbers of closely spaced microelectrodes, such systems make it possible to record activity of large neuronal populations with resolution of individual neurons, providing new insights into processing and coding of information in the brain circuits. Systems with several hundred to a few thousand of channels are now routinely used for recording the brain activity in live animals [8,9] and a prototype device with tens of thousands of recording channels was reported recently [10]. Systems dedicated to large-scale recording of brain activity in human are also being developed [11].

The neural signals acquired by extracellular electrodes are of two types. First, the action potentials (APs) can be recorded from individual neurons located close to the sensing electrode. An action potential is generated by a neuron when the total input signal received by this cell—either from sensory circuits of the central nervous system like the eyes or ears, or from other neurons—exceeds a specific threshold [12]. The APs recorded by extracellular electrodes have forms of short pulses with frequency spectrum from 300 Hz to 5 kHz and amplitudes range 50 μV_pp_–2 mV_pp_ (peak-to-peak). Second, the electrodes can record local field potentials (LFPs) that are primarily generated by ionic currents that occur at the synapses—the physiological connections between neurons—when the information is transferred between cells; however, other processes also contribute to the LFPs [13]. The LFPs are low-frequency oscillations (1–300 Hz) with amplitudes up to 10 mV_pp_.

The readout electronics for modern multielectrode systems is routinely designed as application specific integrated circuits (ASIC) that can comprise hundreds or thousands of recoding amplifiers on a single chip. High-fidelity recording of neuronal signals requires that the noise within the AP range (300 Hz–10 kHz) and the LFP range (1–300 Hz) is not much higher than 5 μV. Also, signals up to ~10 mV_pp_ should be recorded with the total harmonic distortion on the order of 1% or lower [14]. Minimization of the dissipated power and silicon area is also critical for the design of neuronal interfaces with very large number of recording channels.

**Figure 1:**
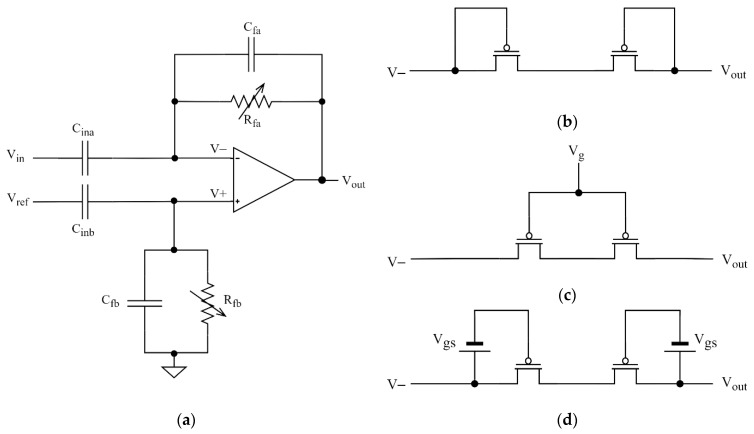
Architecture of an AC-coupled neural amplifier: (**a**) Schematic (**b**–**d**) Implementations of the pseudoresistor: (**b**) diode-connected; (**c**) subthreshold with variable *V_gs_*; (**d**) subthreshold with fixed *V_gs_*. For the following sections we impose *C_ina_ = C_inb_, C_fa_ = C_fb_, R_fa_ = R_fb_*. The distinction between the names of elements of identical values is important for some of the following analyses, where impact of individual elements on the system performance must be analyzed separately.

One technical difficulty in electrical recording of neural signals is related to the large DC voltage at the input of the amplifier that results from electrochemical interactions between the electrode and the tissue [15]. The recording circuit must cut off this DC electrode voltage with high-pass filter with lower cutoff frequency typically on the order of 1 Hz and amplify the remaining AC signals with a gain on the order of 40 dB. Most of the multichannel integrated neural amplifiers are based on the architecture proposed in [15] (Figure 1a); in some designs, the circuit is followed by another amplification stage. The gain of the circuit shown in Figure 1a is given by the *C_ina_/C_fa_* ratio and the cutoff frequency of the AC-coupling circuit is defined by the *R_fa_ × C_fa_* product [15].

Due to silicon area restrictions, the *C_in_* is typically in the range 5–20 pF and the *C_f_* capacitance is typically in the range of tens to hundreds of fF. The feedback resistance in the TΩ range is necessary to achieve sufficiently large time constant. Such a resistance is realized by transistors connected in diode configuration [15] (Figure 1b) or in subthreshold mode that allows one to tune the channel resistance value by changing the gate voltage [16,17,18] (Figure 1c). Such tuning allows the user to control the cutoff frequency of the high-pass filtering and to find—for specific experimental conditions—the optimal compromise between the two requirements:

- efficacy of filtering out of the ultra-slow oscillations present in the brain [19] and electrode drifts, which improves with increasing the cutoff frequency,
- the quality of recording of low-frequency signals with minimized amplitude and phase distortions related to analog high-pass filtering, which improves with lowering of the cutoff frequency.

As the two requirements mentioned above are difficult to define quantitatively in a general way, the ability to control the cutoff frequency of the high-pass filter is a desirable—even if not mandatory—feature of a neural amplifier.

The circuitry shown in Figure 1a makes it possible to achieve very low input-referred noise values. Since the resistor *R_fa_* is in the feedback loop, its thermal noise is divided by the amplifier gain when referred to the input. Although this does not apply to resistor *R_fb_*, its integrated thermal noise is minimized thanks to the parallel connection with the large capacitor *C_inb_* (detailed analysis of the thermal noise introduced by the AC-coupling circuit is presented in Section 4).

Three alternative approaches to remove the electrode offset have been proposed. First, a high-pass RC filter can be used to remove the DC voltage at the input of the amplifier that works without feedback loop [14,20]. This solution has worse noise performance than the circuit shown in Figure 1a, since the thermal noise source from the large resistance is located directly at the amplifier input. The rms (root mean square) value of noise can be minimized by setting the cutoff frequency to very low values—much below the frequency range of interest [20]—but this would compromise on filtering out very slow signals, as discussed previously. As a result, open-loop neural amplifiers use larger input capacitors (20 pF and more) to improve the signal-to-noise ratio (SNR). This comes at the cost of increased area, which is not optimal for systems with high channel counts.

Chopper stabilization is another technique to remove electrode offset in neural amplifiers [21,22]. This method uses modulation to shift the spectrum of the input signal to higher frequencies and to minimize the problem of 1/*f* noise in the amplification circuits [23]. However, high complexity of the design (further increased by a dedicated feedback loop to boost the input impedance, which is low in such circuits) results in increased circuit area.

The third alternative approach to remove the electrode DC voltage is to use a low‑pass filter in the feedback loop of the amplifier, which allows for subtraction of the low-frequency signal components (including the offset) from the input signal by a differential amplifier [24,25]. The low-pass filter requires using additional operational amplifier in the feedback loop that increases the circuit area and power. Using a sigma-delta modulation for low-pass filtering was proposed in [25] with a promise of reduced circuit area in case of using higher-density CMOS process. However, the presented amplifier design in 130 nm technology has a total area (amplifier + ADC) per channel at ~0.05 mm^2^, which is 3–4 times more than the most area-efficient existing designs. At the same time, using very high-density technologies for a large-scale design (with thousands of recording channels) that is not expected to be produced in high volume is impractical because of very high cost of fabrication.

In overall, the architecture shown in Figure 1 remains the gold standard for modern neural amplifiers [26,27,28]. Many designs based on this circuit idea, with excellent noise performance and low power consumption, have been reported; for a review see [29,30]. However, a weak point of the pseudoresistor is its poor linearity. The pseudoresistor is placed in the feedback loop and the voltage drop across this resistance is identical to the amplifier output voltage. Within the range of the output voltage swing (several hundreds of mV to 1 V) the effective resistance of a pseudoresistor may differ by several orders of magnitude [15]. For the circuit with tunable cutoff frequency (Figure 1c) the main reason for the nonlinearity is the modulation of *V_gs_* bias voltage by the continuously changing voltage across the resistor. This nonlinear behavior affects the THD of the circuit, in particular for input signals of large amplitudes and frequencies close to the cutoff frequency of the AC-coupling circuit.

Very few of the published articles on neural amplifiers discuss this problem. Kassiri et al. [31] analyzed several subthreshold two-transistor configurations with fixed-*V_gs_* voltage for the pseudoresistors and achieved significant reduction of nonlinear distortion compared with the classic diode-based architecture. However, the distortion level reported in that paper is still high for 0.5 V_pp_ voltage swing across the resistors. Another paper by the same group includes measurements of the THD vs. signal frequency for the AC-coupled amplifier. The reported value was 3% for the signal frequency equal to cutoff frequency, and input signal amplitude of 1.4 mV_pp_ [32]. The distortion for larger amplitudes was not shown. To our knowledge, this is the only published measurement of THD as a function of signal frequency for amplifiers of this class.

It is important to note that the THD value of neural amplifiers is typically reported in the literature for the frequency of 1 kHz. Since this frequency is about three orders of magnitude higher than the cutoff frequency of the AC coupling circuit, the impedance of the feedback loop at this point is entirely defined by the capacitor *C_fa_*. The THD value defined this way describes the performance of the operational amplifier used for the circuit but—as we show in this paper—it is not related to the distortion produced by the pseudoresistors at low frequencies. To our best knowledge, there has been no multichannel AC-coupled neural amplifier described in the literature with low distortion (~1% THD or less) reported consistently for the complete range of frequencies of extracellular neuronal signals.

In this paper we discuss an improved tunable AC-coupling architecture for CMOS neural amplifiers, based on pseudoresistors built from transistors working with fixed gate-source voltage, which yields low-distortion (~1%) for input signals ranging from 1 Hz to 10 kHz and with amplitudes up to 10 mV_pp_. Based on numerical simulations we describe in detail the mechanism of nonlinear distortion generation and scaling of the THD with the amplifier gain, the cutoff frequency setting, and sizing of the feedback transistors and input capacitors. We also discuss the impact of the AC coupling circuit parameters on the noise performance of the recording system and the noise-distortion design trade-off. Finally, we present results of post-layout simulations of an area-efficient neural preamplifier in an 180 nm PD-SOI technology for verification of the proposed AC coupling architecture at the level of complete integrated circuit.

## 2. Materials and Methods

The work reported here is based on numerical simulations performed in Cadence Virtuoso 6.1.6 (Cadence Design Systems, San Jose, CA, USA). The design has been implemented in an 180 nm PD-SOI technology from XFAB (X-FAB Semiconductor Foundries GmbH, Erfurt, Germany) and all simulations have been performed for the selected technology. Also, for comparison of the key circuit parameters simulations have been performed for other processes (65 and 350 nm). The data analysis and visualization were done using MATLAB (MathWorks, Natick, MA, USA).

### 2.1. Circuits Used for Simulations

To identify the dominant sources of noise and distortion in the circuit and to optimize the design of the proposed amplifier, the analysis of the circuit has been performed in four stages: (a) using a schematic based on ideal passive components (resistor and capacitors) and an ideal differential amplifier, (b) using a transistor level models for the pseudoresistor and an ideal differential amplifier, (c) adding a transistor level models for the amplifier, and (d) using the post layout extracted circuit including parasitic components.

For the analysis of noise contribution of the AC-coupling circuit (Section 4) we used a simplified version of the schematic presented in Figure 1a, with the pseudoresistors replaced by ideal resistors. This allowed us to analyze the thermal noise of resistors *R_fa_* and *R_fb_* separately from other noise sources of the pseudoresistors—like the flicker noise—which could be dependent on technology and sizing of pseudoresistors. For all the other simulations of noise and nonlinear distortion discussed in the paper, the pseudoresistors built from PMOS transistors were used.

For the analysis of nonlinear distortion and noise introduced by the pseudoresistors (Section 3 and Section 4) we used the schematic shown in Figure 1a, with ideal operational amplifier. This allowed us to analyze signal distortions introduced by the pseudoresistor independently of distortions caused by the operational amplifier. The open-loop gain of the operational amplifier was set to 100 dB; increasing the gain beyond this value did not affect the simulation results.

For simulations discussed in Section 6 we used the operational amplifier designed in 180 nm PD-SOI technology. The analysis of nonlinear distortion was based on circuits extracted from the chip layout. For the Monte Carlo simulations, we combined post-layout extracted circuits of a single channel with schematics of all the off-channel circuits to reduce the computational cost of simulations. We confirmed that results of such simulations for nominal circuits (without Monte Carlo sampling) were undiscernible from simulations based on chip-scale extracted circuits.

### 2.2. Methodology of I-V Characteristics Analysis

To characterize linearity of the I-V characteristics of pseudoresistors (Section 3.1) we performed DC simulations. One terminal (named *V-* in Figure 1c,d) of the pseudoresistor was grounded, and the other terminal (named *V_out_* in Figure 1c,d) was connected to varying voltage. Both polarities of the changing voltage were analyzed. For the variable-*V_gs_* configuration the gates of both transistors were shorted and connected to voltage fixed at a constant value with respect to ground. For the fixed-*V_gs_* configuration the gate-source voltages were applied using DC voltage sources as shown in Figure 1d.

### 2.3. Methodology of THD Analysis

All the analyses of the nonlinear distortion of the AC-coupled amplifier were based on transient simulations. We routinely analyzed the THD as a function of the input signal frequency. We ran the simulation separately for every frequency of the input signal within the frequency range of interest. For each simulation the input signal was a stationary sinewave. The THD numbers were calculated offline by taking rms value of the first five harmonics.

We used 15 frequencies points per decade for majority of the performed analyses of THD. For the Monte Carlo analyses we used 5 frequencies points per decade. Initial analyses of distortion (Section 3.2) and final verification of the preamplifier (Section 6) were performed in a wide frequency range (0.1 Hz–10 kHz). For detailed discussion of the distortion introduced by the pseudoresistors we present the results limited to frequency range 0.1–10 Hz where these distortions are most prominent. For plots combining the THD and noise characteristics we use range 0.1–100 Hz to show critical features of all these curves. For plots of the THD we used a piecewise cubic hermite interpolating polynomial (PCHIP) interpolation [33].

With exception of the initial analysis presented in Section 3.2, we used signal amplitude of 10 mV_pp_ for all the simulations of nonlinear distortion. This is the worst case scenario from the distortion point of view, as this is the largest realistic amplitude of the input signal, and the THD values for the analyzed circuit consistently increase with the signal amplitude (Section 3). We note that in experimental practice the recorded signals may have lower amplitudes, in particular at high frequencies. However, the goal of this study is to propose the circuit architecture that is relevant for high-fidelity recording of a complete range of neuronal signals, including large-amplitude voltage oscillations.

### 2.4. Methododology of Noise Analysis

The analysis of the equivalent input noise of the AC-coupled amplifier were based on noise simulations. We routinely analyzed the output-referred noise spectrum and amplifier’s closed loop gain as a function of the input signal frequency. The input referred noise was integrated within three ranges: from 1 to 300 Hz (LFP range), from 300 Hz to 10 kHz (AP range) and from 1 Hz to 10 kHz (full range).

## 3. Amplifier Architecture and Sources of Nonlinear Distortion

### 3.1. DC Analysis of Linearity of Pseudoresistors

As discussed in Section 1, one critical problem with tunable AC-coupled neural amplifier designs in CMOS technology (shown in Figure 1c) is the poor linearity of the pseudoresistors. The main reason for this poor linearity is the variability of the gate-source voltage of the transistors forming pseudoresistors when AC signal is amplified by the circuit. We compared linearity of the I-V characteristics for two configurations of pseudoresistor: a standard configuration based on two PMOS transistors in symmetric configuration (Figure 1c) and alternative configuration with transistors working with fixed-*V_gs_* voltage (Figure 1d) using DC simulations. All the transistors had identical dimensions (*W/L* = 1 µm/40 µm). The small-signal resistances for both pseudoresistors were identical.

**Figure 2:**
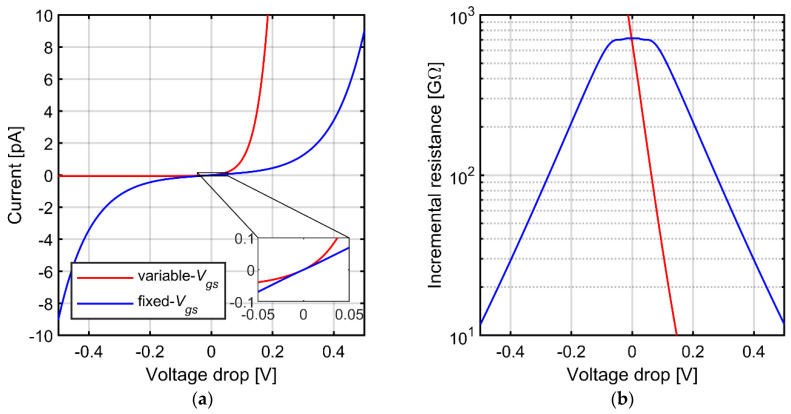
(**a**) Simulated current-voltage relationship for variable-*V_gs_* and symmetric fixed-*V_gs_* pseudoresistors (**b**) Incremental resistance for both implementations.

Figure 2a shows the I-V characteristics and Figure 2b shows the incremental resistance values for both solutions. The linearity of the fixed-*V_gs_* configuration is much better and particularly good within ±100 mV range of voltage across the resistor. We therefore propose to use the fixed-*V_gs_* configuration for the low-distortion amplifier design. We note that such a circuit can be practically realized by including only a single resistor to the amplifier circuit (and additional off-channel circuitry that can be shared by multiple channels), and the required value of this resistor (several hundred of kΩ) means it can be realized in typical CMOS process with small and perfectly linear polysilicon resistor. The complete design of such circuit is presented in Section 6.

Having in mind that the designed circuit aims at low-distortion recording of signals up to 10 mV_pp_, we propose to take advantage of very good linearity of the fixed-*V_gs_* pseudoresistor in the ±100 mV range of the output voltage and to set the amplifier gain value *K* = 20 V/V. We use this value as the nominal gain setting for the following sections, although we analyze impact of the gain on the noise performance (in Section 4) and on distortion (in Section 5). To achieve the required gain of the complete recording circuit (~100) we plan to use a second amplification stage following the presented preamplifier. The second stage can be DC coupled to the output of the preamplifier to avoid additional nonlinearity. The contributions of the second amplifier stage to both noise and distortion of the recording circuit would be much lower than that of the preamplifier and are not analyzed in this paper.

### 3.2. THD-vs-Frequency Characteristics of AC-Coupled Amplifier with Tunable Cutoff Frequency

It is expected that the large difference in the linearity of the I-V characteristics for the variable-*V_gs_* and fixed-*V_gs_* pseudoresistor configurations is reflected in the nonlinear distortions of the complete AC-coupled amplifier processing an AC signal. In particular, one can suspect that highly nonlinear I-V characteristic for the variable-*V_gs_* pseudoresistor configuration should result in very high distortion, making this configuration impractical for amplification of signals up to 10 mV_pp_. To evaluate these effects, we used Spectre transient simulations (Cadence Design Systems, San Jose, CA, UAS) to find the THD-vs-frequency curves for the AC-coupled amplifier (Figure 1a) using the standard tunable and improved tunable pseudoresistor configurations (Figure 1c,d, respectively). The operational amplifier and capacitors *C_in_* and *C_f_* used in these simulations are ideal elements, therefore the only source of nonlinearity is the pseudoresistor in the feedback loop. Transistor models for 180 nm PD‑SOI technology were used for the pseudoresistor.

**Figure 3:**
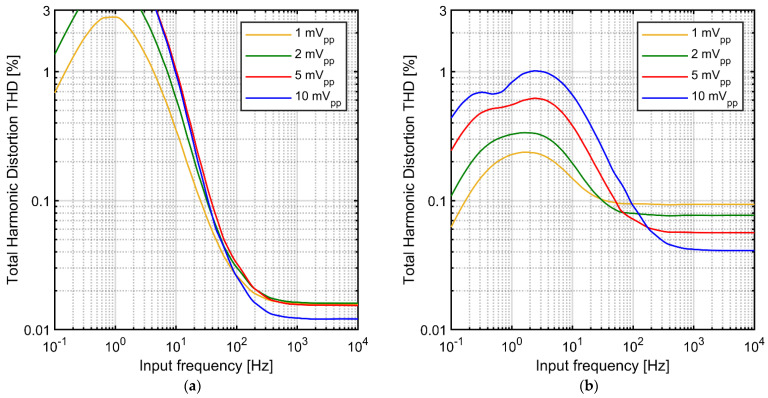
Simulated THD vs. signal frequency for AC-coupled neural amplifier (with 1 Hz cutoff frequency) and for various input signal amplitudes: (**a**) using the variable-*V_gs_* pseudoresistor (**b**) using the symmetric fixed-*V_gs_* pseudoresistor.

The results are presented in Figure 3. We present the THD values in the range limited to 3%—we assume that higher THD values disqualify given curve from further analysis, so the corresponding fragments of the THD curves are not shown to avoid confusion. The THD values for the variable-*V_gs_* pseudoresistor configuration almost reach the 3% threshold already for 1 mV_pp_ signal amplitude (Figure 3a). The shapes of the THD curves for this configuration exhibit a single maximum nearby cutoff frequency of the AC‑coupling circuit. This is easily explained by the increase of the I-V curve nonlinearity for larger voltage drop across the resistor (Figure 2). On one hand, for frequencies below the cutoff frequency of the AC-coupling circuit the signal amplification is reduced which implies lower THD values. On the other hand, for signal frequencies much above the cutoff frequency the impedance in the feedback loop is dominated by the *C_fa_*, therefore the THD values are also lower than for frequencies closer to the cutoff frequency. Combination of these two effects results in the single-maximum shape of the THD curve. The overall increase of distortion for larger signal amplitudes (Figure 3a) is also an expected consequence of highly nonlinear I-V characteristic for large voltages (Figure 2).

We note that although these results clearly show that the variable-*V_gs_* pseudoresistor configuration is not compatible with design requirements for the neural amplifier considered in this paper (signal amplitudes with frequencies down to 1 Hz and amplitudes up to 10 mV_pp_) it may be a reasonable option for different applications. For example, if the recording of low-frequency LFPs is not critical, the cutoff frequency of the AC-coupling circuit can be increased. Since the LFPs amplitude follows, in general, the 1/*f* dependence [19] the signal amplitude in the critical frequency range—that is, close to the cutoff frequency—would be lower than 10 mV and could be amplified with low distortion.

Another potential application is in experiments performed in-vitro, where signals from slices of neural tissue or dissociated neuronal cultures are acquired. As the LFPs produced by such neuronal populations have much lower amplitudes than that observed in the in-vivo measurements, the variable-*V_gs_* configuration could be a sensible option for the pseudoresistor design. In fact, several designs of CMOS neural amplifiers with variable-*V_gs_* pseudoresistors were reported in the literature [16,34,35] (the details of the pseudoresistor configurations differ between specific designs and the linearity performance may also vary to some degree). That said, for the design requirements considered here, the performance of this configuration with respect to nonlinear distortions is not acceptable.

The levels of THD for the fixed-*V_gs_* configuration of the pseuderesistor are significantly lower than for the standard pseudoresistor, especially in the low frequency range around the cutoff frequency (Figure 3). This is expected considering improved linearity of the I-V characteristic for this configuration (compare Figure 2). However, the THD vs. frequency curves for this configuration show particular profiles with two local maxima, which are clearly separated for the highest input signal level of 10 mV_pp_. To explain this shape one must consider additional source of nonlinearity in the circuit, associated with the gate capacitances of the transistors comprising the pseudoresistors.

### 3.3. Impact of Capacitive Gate Currents of Pseudoresistors on THD

**Figure 4:**
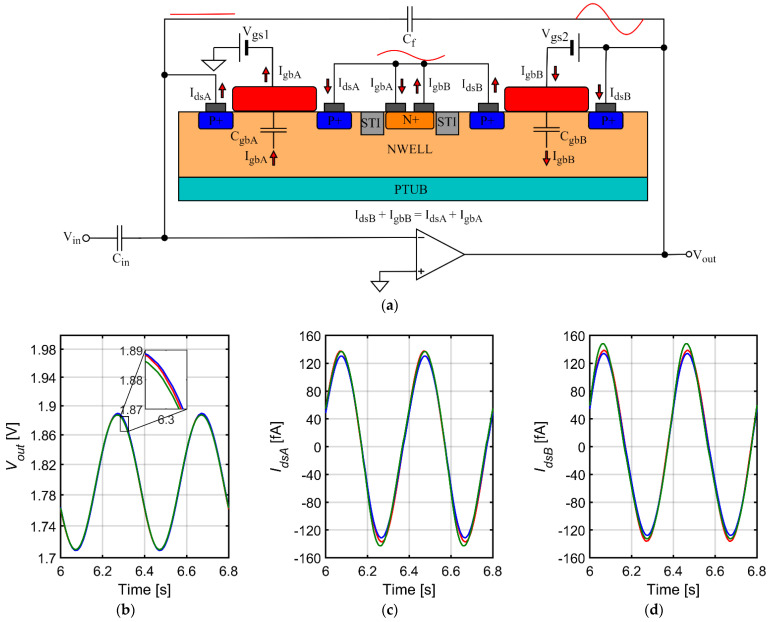

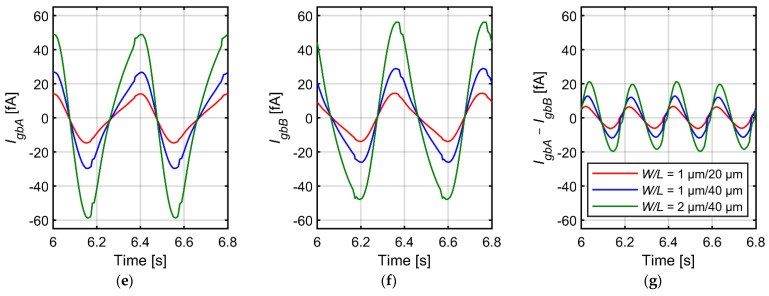
Results of transient simulations for a sinewave signal of 2.5 Hz for three different sizes of transistors composing the pseudoresistor: (**a**) Design of the preamplifier with a model of the fixed-*V_gs_* pseudoresistor; (**b**) Output voltage; (**c**–**f**) Drain-source currents and the gate-bulk currents for transistors A and B; (**g**) Difference between gate-bulk currents for transistors A and B.

A simplified model of the feedback loop for the fixed-*V_gs_* pseudoresistor is shown in Figure 4a. Since the transistors are biased in the deep subthreshold region the gate capacitances are practically equal to the gate-bulk capacitances. These capacitances are not perfectly linear and depend on the *V_gb_* voltage. The AC current *I_gb_* is present in both transistor B (current flow between the output of the amplifier and the common bulk of both transistors) and transistor A (current flow between the common bulk and the external voltage source *V_gs_*_1_—Figure 4a). We note that if the gate-bulk currents were identical for both transistors, all the AC current flowing from the output of the amplifier to the gate of transistor B would go to the external voltage source *V_gs_*_1_. This would be equivalent to additional capacitive load of the amplifier output with no impact on the impedance of the feedback loop, therefore, no impact on THD. However, closer examination of the simulation results shows that the effect is more complex.

In order to confirm that the gate capacitances indeed contribute significantly to the impedance of the feedback loop we show in Figure 4 the results of transient simulations for a sinewave signal of 2.5 Hz for three different sizes of transistors composing the pseudoresistor. The cutoff frequency for each size of transistors was set at 1 Hz by tuning of the *V_gs_* value. The plots show the output voltage, the drain-source currents and the gate-bulk currents for transistors A and B. As expected, the gate currents scale linearly with the gate area. The current is driven by the AC component of the gate-bulk voltage, which is equal to *V_b_–V_ref_* for transistor A and *V_out_–V_b_* for transistor B. Since the *C_gb_* also changes with *V_gb_*, the *I_gb_* is not linear function of *V_gb_* and it must include harmonics of the base frequency of the signal. As visible in Figure 4 the harmonics for transistors A and B are similar in amplitudes but different in phase (Figure 4e,f), and the differential current *I_gbB_–I_gbA_* includes almost entirely the harmonic frequencies (with very low component of the base frequency—Figure 4g). It means that while the base frequency component of the *I_gbB_* current flows in almost 100% through *C_gbB_* and *C_gbA_* to external voltage source *V_gs_*_1_, the current path for the harmonic components of *I_gbB_* closes via the drain-source resistances of transistors A and B. As a result one should expect the increase of the THD values. This effect will be reduced for very low frequencies—where the gate currents become very small compared with drain-source currents—and for frequencies much above the cutoff frequency, where the feedback loop impedance becomes dominated by C_f_. For the amplitude of 10 mV_pp_ the nonlinear gate currents result in second maximum in the THD curve located at ~2.5 Hz and become the dominating source of distortion. For smaller amplitudes these two maxima overlap.

There are two additional issues with the results shown in Figure 3 that require separate comments. First, the nonlinear distortion generation due to AC gate currents, described above, must be also present in the standard variable-*V_gs_* pseudoresistor configuration (Figure 3a). However, with large distortion values caused by the I-V characteristic, this effect is simply too subtle to be visible in the THD plots. Second, the THD curves for the fixed-*V_gs_* pseudoresistor configuration show inversion of the THD vs. amplitude dependence at high frequencies—that is, the THD values decrease when signal amplitude increases. We note, however, that absolute values of the signal harmonics decrease for lower amplitudes. These values are in sub-microvolt range (when referred to the input) for signal amplitude 1 mV_pp_, and will be even lower for smaller signals. Therefore, the effect has no practical implications and is not analyzed in more details here.

### 3.4. Scaling of THD with Gate Area and Oxide Thickness of Transistors Forming Pseudoresistors

**Figure 5:**
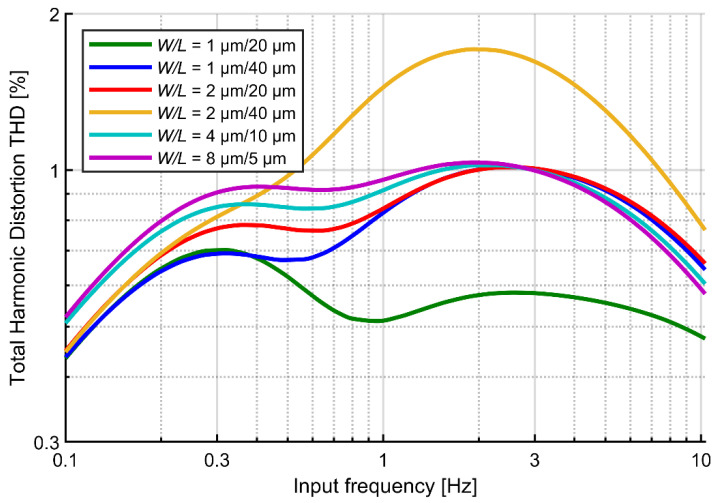
Simulated THD versus signal frequency for the fixed-*V_gs_* circuit for different dimensions of the pseudoresistor. Signal amplitude: 10 mV_pp_. *V_gs_* values were tuned for each simulation to get the same cutoff frequency (1 Hz).

The contribution of gate current to the circuit distortion is expected to scale with the gate area of transistors A and B. Figure 5 shows the simulated THD value as a function of input signal frequency for various sizing of the transistors and large signal amplitude (10 mV_pp_). As expected, the height of the second peak (at 2–3 Hz for different curves) scales with the product *W × L*; in contrast, the first peak is associated with the nonlinearity of the I‑V curve (at ~0.3 Hz) scales with the transistor channel length. These observations may lead to a conclusion that reducing the gate area of transistors forming the pseudoresistor is a way for reduction of the THD. However, we note that excessive reduction of the gate areas may result in significant mismatch of transistors A and B and disturbed symmetry of I-V curve for positive and negative voltages. This would lead to increased THD values. For this reason, we accept the gate width *W* = 1 μm and length *L* = 40 μm for the following analyses.

**Figure 6:**
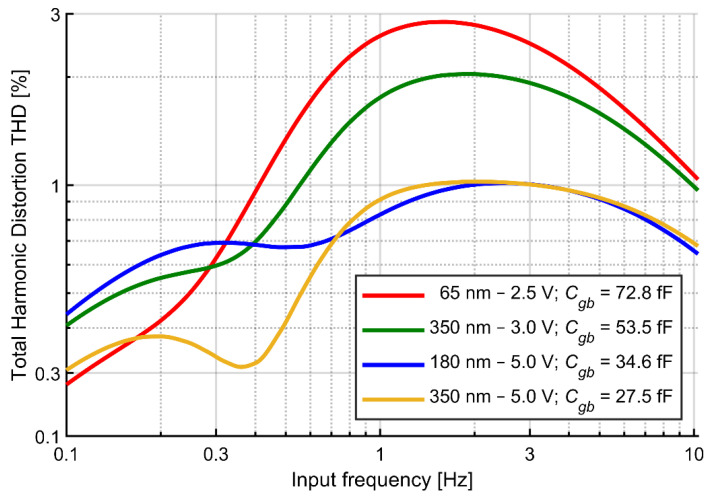
Simulated THD versus signal frequency for the fixed-*V_gs_* circuit for different technologies. Signal amplitude: 10 mV_pp_. *V_gs_* values were tuned for each simulation to get the same cutoff frequency (1 Hz).

Finally, the presented analysis suggests that the thickness of the gate oxide should also affect the circuit distortion. We performed simulations of the circuit shown in Figure 1a with fixed-*V_gs_* pseudoresistors designed in three different CMOS technologies (350, 180 and 65 nm), using the available thick-oxide transistors for the pseudoresistors. The sizes of transistors were identical in all simulations (*W* = 1 μm, *L* = 40 μm) and the *V_gs_* values were tuned for each simulation to get the same cutoff frequency (1 Hz). The results are shown in Figure 6. Consistently with the analysis presented above, transistors with larger oxide thickness (and proportionally lower gate-bulk capacitances) yield lower distortion related to gate-bulk currents. The 5 V transistors in two technologies (350 and 180 nm) result in virtually identical THD values above the cutoff frequency. Below the cutoff frequency, where THD value is determined by the nonlinearity of the I-V characteristic, the distortion does not correlate with the oxide thickness. Although the results suggest that technologies that provide higher voltage transistors (5 V) may be preferable, we note that the distortion also critically depends on the gate area. Since more advanced technologies offer in general improved transistor matching [36] it may be possible to use smaller transistors in more advanced nodes to compensate for lower oxide thickness. These aspects require further systematic studies. For this work, the 180 nm technology with 5 V transistors for pseudoresistor design was chosen.

## 4. Noise Contribution of the AC-Coupling Circuit

Since a neural amplifier must be capable of recording signals with amplitudes down to tens of µV with good SNR, noise performance is a critical aspect of the design [15]. In this context, the noise contribution of the resistive elements of the AC coupling circuit must be carefully analyzed.

The results presented in this section are based on simulations of circuit shown in Figure 1a, with ideal operational amplifier and ideal resistors. This way, the thermal noise of the resistors is analyzed in separation from other sources of noise associated with the pseudoresistors (like flicker noise) or with the operational amplifier. In the Section 5 we discuss noise and nonlinear distortion based on simulations taking advantage of pseudoresistor built from PMOS transistors.

We start with analyzing the noise contribution from the feedback resistor *R_fa_* (compare Figure 1a). At this point we assume the non-inverting input is at virtual ground, therefore the noise measured across the *R_fa_* is equivalent to the noise contribution of this resistor measured at the output of the circuit. The total rms voltage noise of this resistor is:

(1) $$V_{ni,rms\;R_{f}}=\sqrt{\frac{kT}{C_{fa}}},$$

and is independent of the *R_fa_* value and the cutoff frequency. However, this value comes from integration of power spectral density (PSD) curve from 0 to infinity. In order to quantify noise level in the defined frequency range, we need to look closer at the noise spectrum.

**Figure 7:**
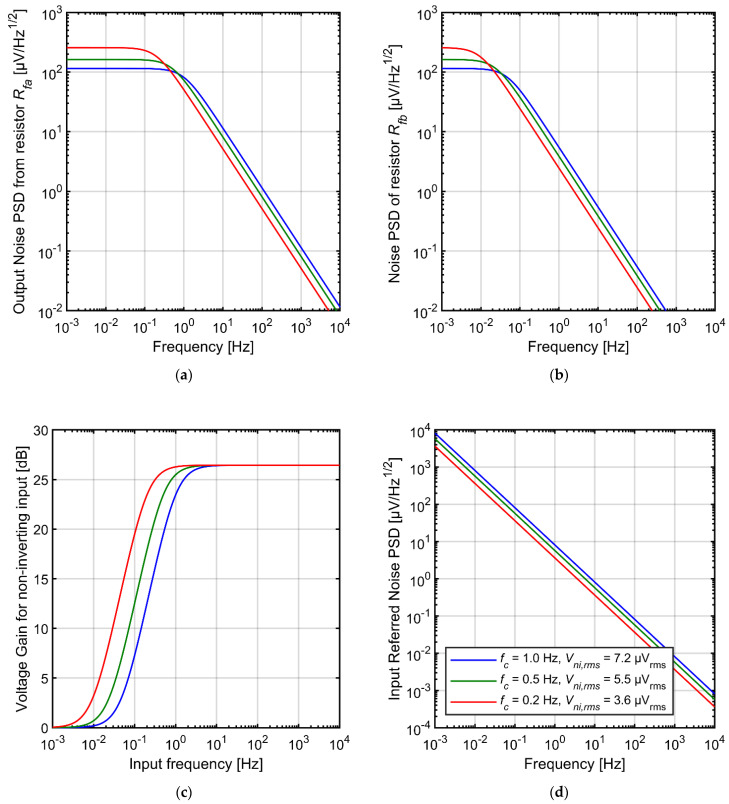
Characteristics of the AC-coupling circuit thermal noise for different settings of the AC-coupling circuit cutoff frequency (**a**) output noise PSD from resistor *R_fa_* (**b**) noise PSD of resistor *R_fb_* measured across this resistor (**c**) AC gain of the circuit with respect to non-inverting input of the operational amplifier (**d**) PSD of the combined input-referred noise from both resistors *R_fa_* and *R_fb_*.

In Figure 7a we present the PSDs of the noise contributed by *R_fa_*. The PSDs are shown at the amplifier output for various values of the cutoff frequency of the AC-coupling circuit. Although the tuning does not change the total rms noise, it shapes the noise spectrum. Therefore, the noise in the frequency range of interest can be reduced by shifting the cutoff frequency below this range; this is discussed in more detail later in this section.

In order to explain the noise contribution of the resistor *R_fb_*, we analyzed the PSD of its thermal noise (measured across the resistor itself) and the transmittance of the complete circuit with respect to signals that appear at the noninverting input of the operational amplifier U1 (compare Figure 1a). The plots are shown in Figure 7b,c. Since the resistor *R_fb_* is shunted with large capacitance (*C_inb_ + C_fb_*) the PSD values for most of the frequency range are much lower than those shown in Figure 7a. On the other hand, the 1/*f* dependence is extended toward lower frequencies as the time constant has a large value of *R_fb_ × (C_in_ + C_fb_)*. For extremely low frequencies (below 0.1 Hz) the PSD values of the curves shown in Figure 7a,b become identical. At the same time, the circuit transmittance for this noise is equal to 1 at the very low frequencies and equalizes at value *(C_ina_ + C_fa_)/C_fa_* for higher frequencies. Multiplication of the respective curves shown in Figure 7b,c results in the output noise PSDs characteristics that are identical to that presented in Figure 7a. We conclude that the resistors *R_fa_* and *R_fb_* have identical impact on the noise performance of the circuit, both in terms of noise PSDs and the rms values.

The input-referred noise rms values calculated in 1 Hz–10 kHz frequency range are given in Table 1. One way to reduce the noise contribution of the AC-coupling circuit in specific frequency range is to set very low cutoff frequency. Some designs take advantage on this by shorting the gate and the source of the pseudoresistors, which leads to very large resistances of *R_fa_* and *R_fb_*. Such a solution will result in extremely low cutoff frequency in the range of tens of mHz or even lower and greatly reduced noise from feedback resistors above 1 Hz. Unfortunately, such filters do not remove the very slow and large-amplitude drifts of the electrode voltage from the signal, and this can lead to saturation of the recording amplifier. For this reason some users prefer circuits with tunable cutoff frequency which is set closer to the frequency range used for analyses, as discussed in the Introduction. Nevertheless, careful optimization of the filter time constant for specific experimental conditions may potentially be very useful in reducing the system noise.

The noise can be also reduced by decreasing the feedback capacitance *C_fa_* and consequently increasing the preamplifier gain (Table 1). We speculate that for this reason virtually all the reported CMOS neural amplifiers use higher gain of the first amplification stage than the circuit proposed in this work. However, as increasing the gain results in higher voltage drop across the feedback resistor *R_fa_*, it is expected that the side effect of such a solution will be higher signal distortion, particularly for large input signals. We provide more detailed discussion on distortion-vs-noise trade-off in Section 5.

Finally, an easy way to reduce the noise level is to increase the values of capacitors *C_ina_*, *C_inb_*, *C_fa_* and *C_fb_* (Table 1). Unfortunately, this comes at the price of circuit area. In reality, the total silicon area of many reported neural amplifiers is primarily defined by the capacitors—namely, both the input capacitors *C_ina_* and *C_inb_* (Figure 1). For design aiming for a compact amplifier footprint the capacitances must be kept as small as possible.

The analysis presented in this section and the noise values given in Table 1 lead to conclusion that that the proposed design parameters (*C_ina_ = C_inb_* = 4 pF, *C_fa_ = C_fb_ =* 200 fF, *K* = 20) should allow for reasonably good noise performance, comparable with advanced multichannel neural amplifiers reported in the literature [10,28]. At the same time, the moderate gain value *K* = 20 V/V should help keeping the level of distortion under control. We therefore accept these parameters as the starting point for the following detailed analysis of the circuit distortion.

## 5. Design for Low Distortion, Low Noise and Small Silicon Area

In Section 3 we described the mechanism of nonlinear distortion generation based on simulations of the circuit with nominal settings (*K* = 20 V/V, *C_in_* = 4 pF, cutoff frequency = 1 Hz). In this section we analyze how changing of these parameters affects the THD values and we discuss the distortion-noise trade-off. The results are based on simulations with an ideal operational amplifier so we can analyze distortion and noise introduced by the pseudoresistor decoupled from nonidealities of the operational amplifier itself. The pseudoresistors in these simulations are built from 5 V PMOS transistors (*W/L* = 1 μm/40 μm). We note that the results of the noise analyses presented here may be slightly different than those presented in Section 4, where only the thermal noise from an ideal resistor was considered; however, the conclusions given in Section 4 are sufficient to explain qualitatively the results presented here.

**Figure 8:**
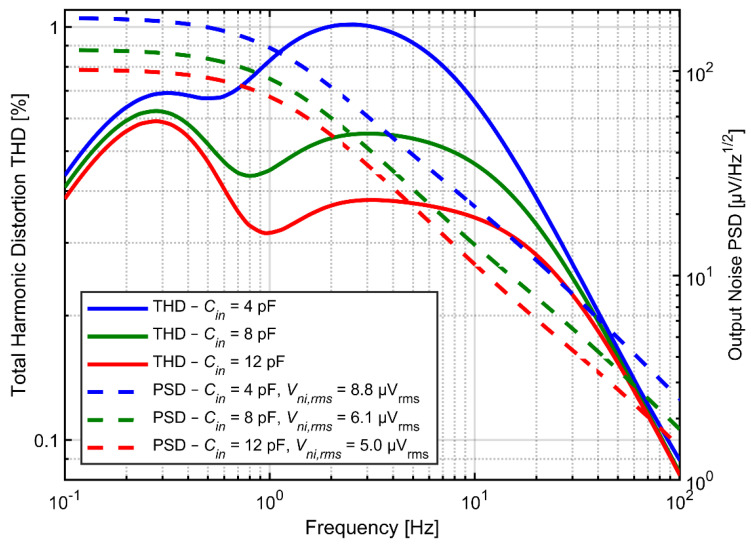
THD as a function of input signal frequency and output noise PSD for various values of *C_in_* and *C_f_* with fixed ratio *C_in_/C_f_* = 20. Cutoff frequency is fixed at 1 Hz.

Figure 8 shows the noise PSD and the THD as a function of input signal frequency for various values of *C_in_* and *C_f_* but at fixed ratio *C_in_/C_f_* = 20. Both the noise and distortion performance benefit from larger capacitors. Increasing the *C_f_* determines proportional decrease of the feedback resistance if the time constant of the high-pass filter is not expected to change. Obviously, the total current in the feedback loop increases proportionally to the increase of capacitors. The same applies to current in capacitor *C_fa_* as well as to the drain-source currents of transistors forming the pseudoresistor (the ratio of drain-source current to current in *C_fa_* for given frequency does not change, since we assumed that the time constant did not change). At the same time the gate currents for transistors A and B (Figure 4a) do not change (as both the gate capacitances and the gate-to-bulk voltages for these transistors do not change). In consequence the influence of the current *I_gbB_–I_gbA_* on the drain-source currents is reduced and the second peak in THD curves decreases, according to analysis presented in Section 3.3. On the other hand, increasing of *C_fa_* and *C_ina_* results automatically in reduction of thermal noise across both resistors *R_fa_* and *R_fb_*_._

**Figure 9:**
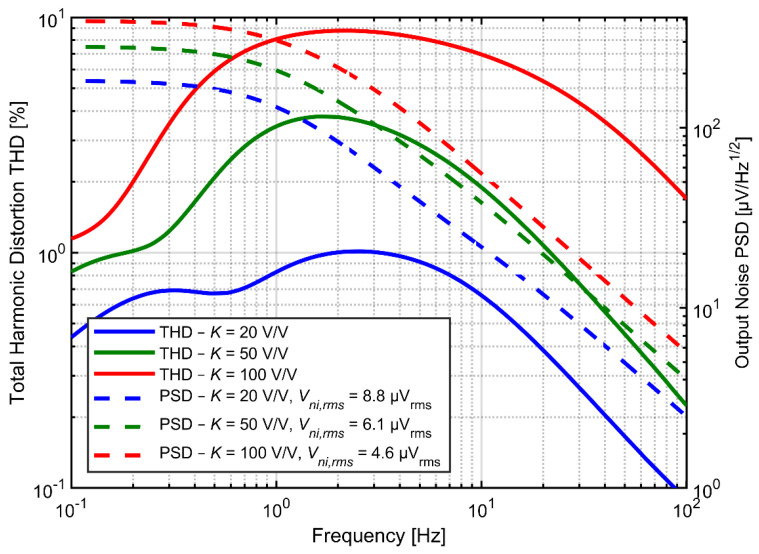
THD as a function of input signal frequency and output noise PSD for various values of *K* and C_f_. *C_in_* value is fixed at 4 pF and cutoff frequency is fixed at 1 Hz.

Figure 9 presents the dependence of THD and noise spectrum on the amplifier gain. For the three gain settings the *C_in_* value was fixed at 4 pF and the *C_f_* value was set to 200, 80 or 40 fF for the gain of 20, 50 and 100 V/V, respectively. On the other hand, larger gain results in significant increase of the distortion, for two reasons. First, increased gain results in larger amplitudes of *V_out_*, for which the pseudoresistor linearity becomes much worse; this effect is visible in the DC I-V curves in Figure 2. Second, the distortion related to gate-bulk nonlinear currents are also expected to increase, since these currents increase accordingly with larger gate-bulk voltages, and the total current in the feedback loop remains the same (as *C_in_* does not change). The end result is that the ratio of *I_gbB_–I_gbA_* current to the drain-source currents is higher, and so are the THD values. On the other hand, since the noise rms is inversely proportional to the square-root of *C_f_* and the gain is inversely proportional to *C_f_*, the input-referred noise from the feedback resistor is lower if *C_f_* is reduced. We conclude that for designs with strict limits on the silicon area, when *C_in_* must be kept small, the gain of the preamplifier should be optimized for specific application in order to get the best compromise between noise and distortion values.

**Figure 10:**
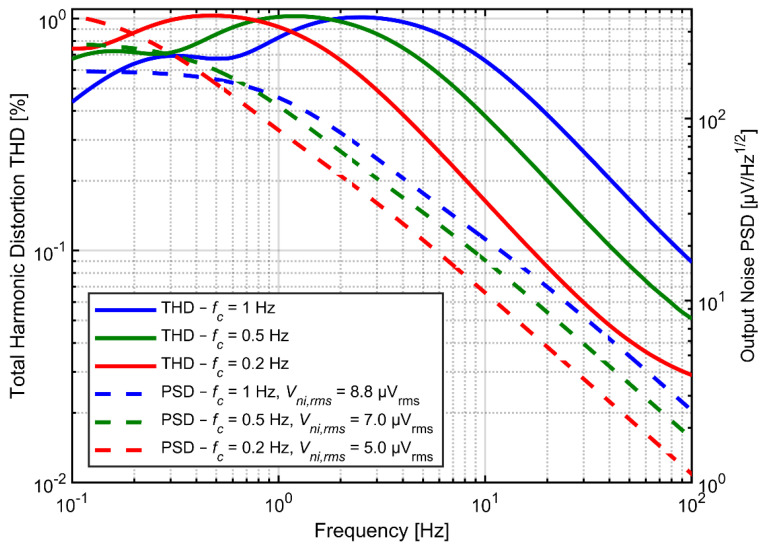
THD as a function of input signal frequency and output noise PSD for various values of the cutoff frequency with *C_in_* = 4 pF and *C_f_* = 200 fF (*K* = 20 V/V).

Tunable AC coupling circuit provides the opportunity to shape the spectrum of noise and distortion. One can shift the maxima of the THD curve out of the frequency range of interest by lowering the cutoff frequency (Figure 10). However, the low frequency signal components can still generate harmonics leaking into higher frequency range and can modulate higher-frequency signals due to circuit nonlinearities. It is therefore difficult to analyze the profit of the cutoff frequency decrease on the output signal distortion without knowing the spectrum of the input signal, and in particular, the power of very slow (out‑of‑band) oscillations of the electrode voltage. The positive effect of lowering the cutoff frequency on the noise measured above 1 Hz is straightforward, as shown in Figure 10.

## 6. Complete Preamplifier Design

**Figure 11:**
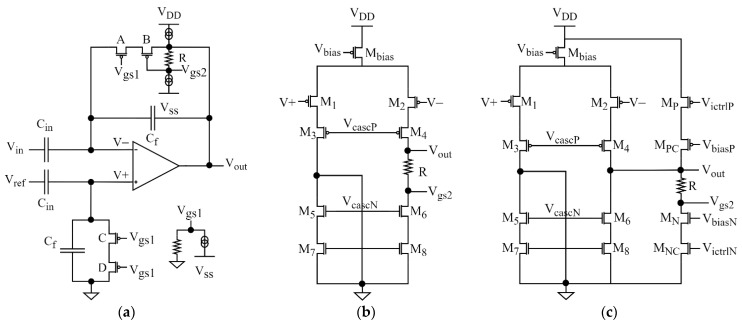
Design of the preamplifier: (**a**) Fixed-*V_gs_* AC-coupling architecture using polysilicon resistors; (**b**) Design of the preamplifier with integrated polysilicon resistor; (**c**) Design of preamplifier for the test chip.

In order to verify results of our analyses in practical circuit we designed a complete neural preamplifier based on the AC coupling architecture discussed above. Figure 11a presents the block diagram of the preamplifier. We note that the *V_gs_* voltage that tunes the feedback resistance is generated differently for transistors A and B. Since the source of transistor A is at virtual ground, the gate potential of transistor A can be generated off‑channel and shared between all the channels of the ASIC.

The gate potential of transistor B must be shifted by a constant value (*V_gs_*) from the output voltage of the amplifier. This can be easily realized by integrating a single resistor into the amplifier, as shown in Figure 11b, and taking advantage of the bias current of transistor M2 to generate the *V_gs_*. The required *V_gs_* values are on the order of 250–400 mV (see below) so assuming the bias current of ~1 µA, the value of resistor R must be in the range of several hundreds of kΩ. Such values can be easily realized using a polysilicon resistors that typically have resistivity of a few kΩ per square (6.6 kΩ per square in case of process used in this work). Due to excellent linearity and matching properties of the polysilicon resistors, the proposed method for generation of *V_gs_* value may be preferable to alternative solutions based on transistor-based voltage shifter [31]. Although tuning of the feedback resistance requires changing of the bias current which affects the thermal noise of the preamplifier (contributed mostly by transistors M_3_ and M_4_ in Figure 11b), this effect is negligible. The cutoff frequency scales exponentially with *V_gs_* and can be shifted by an order of magnitude from its nominal value (1 Hz) with changing the bias current by ~25%. This results in a change of the thermal noise of transistors M_3_ and M_4_ by only ~12%.

For the design of the test integrated circuit we decoupled the controls of bias current and cutoff frequency, as shown in Figure 11c. This will allow us to measure the noise contribution of the amplifier as a function of bias current without changing the cutoff frequency. The current flowing through the polysilicon resistor is generated by cascode current sources. We used the resistor of 1 MΩ and the current of 315 nA is necessary to set the cutoff frequency to 1 Hz. For the design of the preamplifier we used the telescopic cascode architecture. Because the amplifier is designed for bidirectional neural interfaces with electrical stimulation capability, we plan to use relatively high supply voltage (3.0–3.6 V) for which the telescopic cascode architecture offers the best noise/power performance [37] and provides enough voltage headroom for the ±200 mV_pp_ ac voltage swing. The supply voltage for the simulations was set at ±1.8 V with respect to ground.

**Figure 12:**
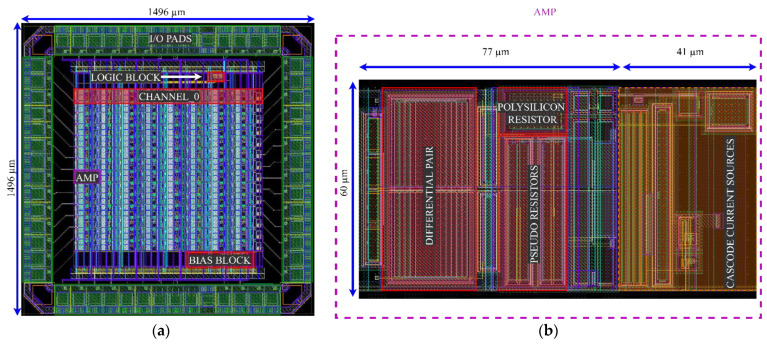
(**a**) Layout of the test integrated curcuit; (**b**) Layout of the preamplifier for the test chip. The elements inside the orange rectangle include the cascode transistors (*M_P_, M_PC_, M_NC_, M_N_*—compare with Figure 11c) and an output buffer; these blocks will not be included in the final preamplifier design shown in Figure 11b. The layout of the preamplifier is 118 μm × 60 μm (77 μm × 60 μm for the final design).

Figure 12 shows the layout of the test integrated circuit. The chip includes 14 identical channels. Each channel includes eight versions of the preamplifier differentiated by the sizing of the pseudoresistors (four versions with *W/L* respectively: 2 μm/40 μm, 1 μm/40 μm, 2 μm/20 μm, 1 μm/20 μm) and capacitors *C_in_/C_f_* (two versions: 4 pF/200 fF and 8 pF/400 fF). The design of the operational amplifier is identical for all 8 versions. The bias current and *V_gs_* are controlled externally. The design was submitted to fabrication and the detailed measurements report will be published separately.

**Figure 13:**
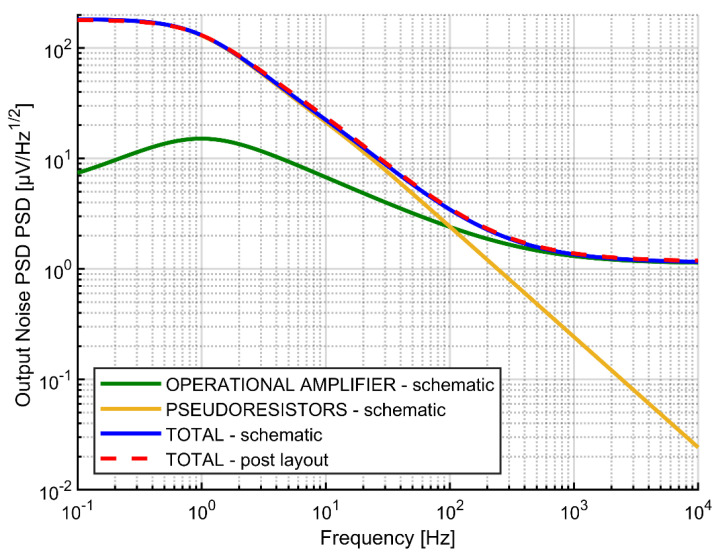
Output noise PSD for pseudoresistors and operational amplifier.

The results of noise simulations are presented in Figure 13 and Table 2. The total noise is dominated by the pseudoresistors in the LFP range (1–300 Hz) and by the preamplifier noise in the AP range (300 Hz–10 kHz). For both frequency ranges the noise on the order of 6 µV_rms_ is achievable. The results of post-layout simulations are perfectly consistent with simulations based on the schematics.

**Figure 14:**
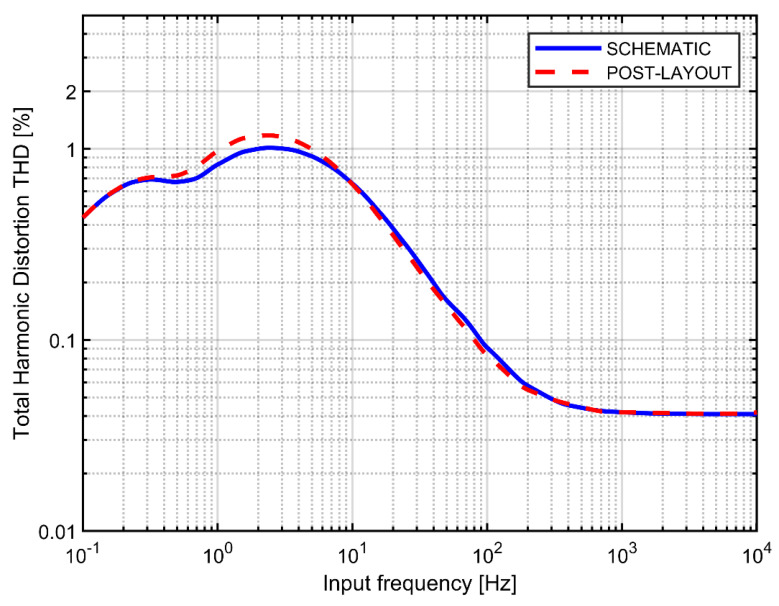
Simulated THD versus signal frequency for the complete AC-coupled preamplifier. Signal amplitude: 10 mV_pp_, cutoff frequency 1 Hz.

**Figure 15:**
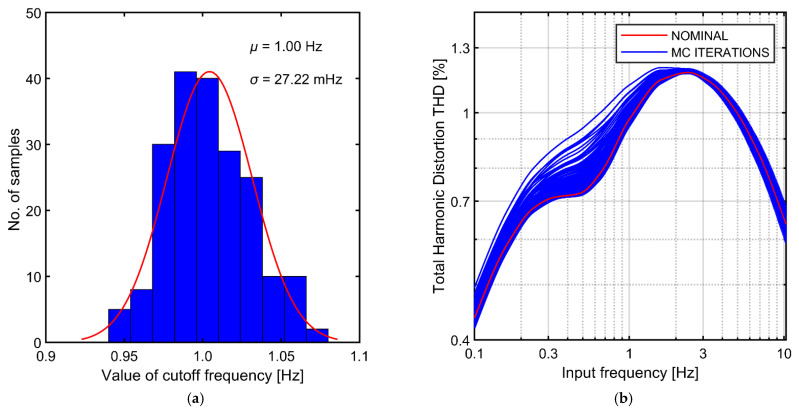
Results of Monte Carlo simulations of complete AC-coupled preamplifier: (**a**) Spread of the cutoff frequency; (**b**) Spread of the THD versus signal frequency characteristics.

In Figure 14 we compare the post-layout simulations of the THD-vs-frequency characteristic of the circuit presented in Figure 11c with schematic-based simulations of the circuit based on an ideal operational amplifier (discussed in the previous sections). The post-layout results show slightly higher THD peak at around 2.5 Hz (1.17% vs. 1.01%); otherwise the two graphs are very similar. Finally in Figure 15 we present the results of post-layout Monte Carlo simulations of the THD curves. The transistors mismatch leads to slight increase of THD below the cutoff frequency, which is associated with perturbed symmetry of the I-V curve for positive and negative voltages. However, the peak at ~2.5 Hz that is responsible for the global maximum of the THD characteristic, is not affected by the mismatch.

The parameters of the test ASIC are given in Table 3. The results suggest that the proposed circuit should be capable of providing the low-distortion amplification of full range of neuronal signals, with competitive noise and power figures and very small design area. However, small corrections of the preamplifier gain and/or absolute values of capacitors *C_in_* and *C_f_* may be necessary to meet the goal of <1% THD value for large signal amplitudes (10 mV_pp_) and across complete range of signal frequencies. These considerations will be concluded based on detailed measurements of the test chip.

## 7. Conclusions

In this article we present an improved AC-coupled CMOS neural amplifier that operates with low nonlinear distortion over wide range of signal frequencies (1 Hz–10 kHz) and amplitudes (up to 10 mV_pp_). Since in the proposed circuit pseudoresistors that control the time constant of AC-coupling circuit work with fixed *V_gs_* voltages, the linearity of the resistance is improved and the THD values are greatly reduced. The proposed solution requires adding of only a single compact polysilicon resistor to the preamplifier schematic. We describe the origins of nonlinear distortion and analyze the THD as a function of signal frequency and amplitude. We also analyze the impact of basic amplifier parameters (silicon area, gain and cutoff frequency) on the distortion and noise performance of the circuit. Post-layout simulations confirm that the proposed preamplifier is suitable for recording the full spectrum of electrophysiological signals with low distortion (THD < 1.17%) and competitive noise performance (~8.3 µV_rms_). Compared with the standard solution using the AC-coupling circuit with variable *V_gs_*, the circuit described here provides reduction of the THD values at low frequencies and large amplitudes by more than one order of magnitude. High-fidelity signal amplification and very compact footprint of the preamplifier (0.0046 mm^2^) make the design relevant for the future CMOS-based very large scale neuroelectronic interfaces.

## Figures and Tables

**Table 1:** Input-referred noise rms for various parameters of the circuit. The rms values are calculated in 1 Hz–10 kHz frequency range. Assumptions: *C_ina_ = C_inb_ = C_in_, C_fa_ = C_fb_ = C_f_, R_fa_ = R_fb_ = R_f_*.

Characteristic Parameters	*C_in_, C_f_* [F]	AC Gain—*K* [V/V]	Cutoff Frequency of the AC-Coupling Circuit [Hz]	Equivalent Input Wide-Band Noise [μV_rms_]
Variable cutoff frequency (variable *Rf*)	4 p, 200 f	20	1.0	7.2
	4 p, 200 f	20	0.5	5.5
	4 p, 200 f	20	0.2	3.6
Variable AC gain (variable *C_f_*)	4 p, 200 f	20	1.0	7.2
	4 p, 80 f	50	1.0	4.6
	4 p, 40 f	100	1.0	3.2
Variable design area (variable capacitors)	4 p, 200 f	20	1.0	7.2
	8 p, 400 f	20	1.0	5.1
	12 p, 600 f	20	1.0	4.2

**Table 2:** Equivalent input noise [μV_rms_]. For each bias current and cutoff frequency the noise is shown for two frequency ranges: 1–300 Hz and 300 Hz–10 kHz.

Cutoff Frequency of the AC-Coupling Circuit	Total Bias Current		
	2 µA	4 µA	6 µA
1.0 Hz	9.16 | 6.18	9.03 | 4.6	9.02 | 3.93
0.5 Hz	7.49 | 6.15	7.29 | 4.57	7.26 | 3.90
0.2 Hz	5.66 | 6.13	5.44 | 4.55	5.41 | 3.87

**Table 3:** Parameter of the amplifier based on post-layout simulations. Cutoff frequency was set at 0.2 Hz.

Parameters	Values
Open loop gain	89.5 dB
AC gain	25.9 dB
Total bias current	2 μA
Power dissipation per channel	7.2 μW
Equivalent input noise in the LFP range	5.66 μV_rms_
Equivalent input noise in the AP range	6.13 μV_rms_
Equivalent input wide-band noise	8.34 μV_rms_
NEF	4.55
Area single amplifier prototype	0.0071 mm^2^
Area single amplifier simplified	0.0046 mm^2^

## Data Availability

Not applicable.
